# Ealuation of Occupational Factors on Continuation of Breastfeeding and Formula Initiation in Employed Mothers

**DOI:** 10.5539/gjhs.v5n6p166

**Published:** 2013-09-25

**Authors:** M. Ahmadi, S. M. Moosavi

**Affiliations:** 1 Community Medicine Department, Medical College, Mazandaran University of Medical Sciences, Sari, Iran; 2 Psychiatry Department, Zare Hospital, Mazandaran University of Medical Sciences, Sari, Iran

**Keywords:** mother milk, employed mother, return to work

## Abstract

**Background and Objective:**

During recent decades, women have been increasingly involved in social activities. Despite the fact that mothers prefer to breastfeed, their return to work is associated with a reduction in breastfeeding frequency and duration. The present study evaluates the impact of occupational factors on continuation of breastfeeding and formula initiation in employed mothers with infants aged 6-12 months in Bandar-Abbas, Iran in 2010.

**Method and Materials:**

This is a descriptive-analytic study on employed mothers with infants aged 6-12 months referring to healthcare centers of Bandar-Abbas in 2010. Data were collected through a questionnaire dealing with work-related factors in mothers’ workplace.

**Findings:**

Out of 212 mothers who responded, 52.38% used formula to feed their children, and 27.36% had discontinued breastfeeding. The rate of formula use was significantly higher in mothers who had less than 6 months of maternity leave, those who did not have a suitable nursery or place to milk themselves and preserve the milk in their workplace, those working more than 6 hours per day, and those who could not take a breastfeeding break.

**Conclusion:**

It is essential to identify and support breastfeeding employed women. The employers should provide facilities such as nurseries, a suitable physical space for milking, as well as the equipment necessary for milk preservation. Also, such mothers should be granted breastfeeding breaks to feed their child or milk their breasts.

## 1. Introduction

The latest surveys in Iran place Exclusive breastfeeding rate in <6 months is 23% (unicef, 2011) and the mean age of breastfeeding discontinuation at 10.7 months According to the Ministry of Health & Medical Education (2005). Studies conducted over the last 10 years mention maternal employment as the most important factor affecting this issue (Breastfeeding guide for employed mothers, 2002; Mohammadi, Dadkhah, & Mozaffari, 2004; Mohsenzadeh & Mardani, 2008; Ghotbi, 2008; Khayyati, 2008; Abedzadeh, Saberi, & Sadat, 2006; Ghaed et al., 2005; Akaberian & Dianat, 2004; Hajian, 2002; Mamoori & Hashemi, 2002). Mother’s employment is the most common cause of mother-infant separation during the breastfeeding stage (Schanler, 2006). The executive mandate of the Law for Promotion of Breastfeeding states that the maternity leave for governmental and private sectors is 6 months. Also, once the mothers return to work, they are allowed a breastfeeding break of an hour each day (three times, each time 20 minutes) until 24 months, and the employers are required to provide the appropriate facilities for their female workers According to the Ministry of Health & Medical Education (2007). Nevertheless, the maternity leave is shorter than legally stated in many private organizations and many of these organizations lack the necessary facilities to encourage breastfeeding According to the Ministry of Health & Medical Education (2005). Early return of mothers to work (especially within 6 months of delivery) has a tremendous impact on continuation of breastfeeding (Chuang et al., 2010; Ogbuana, Glover, Probst, Liu, & Hussey, 2011). Moreover, longer work hours reduce the chance of breastfeeding as well as its duration (Ogbuana et al., 2011). One efficient solution is part-time employment which improves the duration of breastfeeding once the mother returns to work (Mandal, Roe, & Fein, 2010). In addition, educating employers for supporting employed mothers and providing the necessary facilities (e.g. milking room, milking and preservation equipment, and nurseries at workplace) will help resolve this challenge (Amin et al., 2011; Rojjanasrirat & Sousa, 2010; Payne & Nicholls, 2010; Morais et al., 2011). Considering the fact that breastfeeding has an immense impact on the physical and mental health of both infant and mother, as well as the fact that any threats to breastfeeding will compromise the health of families, the community and the healthcare policies of the nation, it appears that investigating the factors affecting breastfeeding rates in employed women and finding solutions will improve the healthcare status in Iran. The aim of the present study is to evaluate the occupational factors affecting continuation of breastfeeding and formula initiation.

## 2. Method and Materials

In a cross-sectional study, 212 mothers who were referred to any of the sixteen healthcare centers of Bandar Abbas for routine infant care (growth monitoring or vaccination) were selected through convenience sampling. The inclusion criteria were: maternal employment, infant aged 6-12 months, infant being healthy (verified through history taking, health certificates, and physical examination by a pediatrician). After providing explanation for the mothers about the study and reassuring them about the anonymity of questionnaires and confidentiality of their information, written informed consents were obtained from them. The questionnaires were completed by a research assistant. The questionnaire was devised using the Breastfeeding Handbook for Physicians published by the American Academy of Pediatricians and the American College of Obstetricians and Gynecologists. The reliability of the questionnaire was confirmed with Cronbach’s alpha (0.77) and its validity with test re-test. The questionnaire consisted of 14 items which inquired about the occupational characteristics of the workplace (related to breastfeeding) using Yes/No questions. The exclusion criteria were using formula for any reason other than maternal occupation, maternal physical or mental illness, special medication used by mother, maternal addiction, multiple pregnancy, adopted child. The sample size was estimated at 212 mothers using the results of previous studies and the sample size equation:


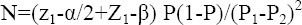


Considering the fact that this is a descriptive-analytic study and no intervention was implemented, the anonymity of questionnaires, as well as the fact that no stage of the study needed the participants’ personal information and they all expressed their informed consent in written, we have abided with ethical considerations throughout the study[MH1]. Once the questionnaires were completed, data were analyzed using chi-square test on SPSS version 20, and p values < 0.05 were considered significant.

## 3. Results

In the present study dealing with the occupational factors affecting the continuation of breastfeeding and formula initiation in employed mothers with infants aged 6-12 months in Bandar Abbas in 2010, a total of 212 cases were evaluated. The mothers were aged 22 to 39 years. 65 mothers (30.66%) were aged 22-27 years, 112 (52.83%) were aged 28-33 years, and 35 (16.51%) were aged 34-39 years. 176 mothers (83.02%) were employed in governmental institutions and 36 (16.98%) worked in the private sector. 112 mothers (52.83%) used formula and 100 (47.17%) managed to continue breastfeeding without using formula. Data on infants’ feeding after mothers’ return to work are presented in Table 1.

**Table 1:** Type of infant feeding after mother’s return to work during mother-infant separation hours

Type of Feeding	Count	Percent
Direct Breastfeeding	90	42.45
Pumped breast milk	114	53.78
Formula	112	52.83
Wet Nurse	10	4.72
Supplemental Food	6	2.83

Among mothers who used formula, 58 (51.79%) had completely stopped breast milk (7.36% of all mothers), and 54 (48.21%) used formula alongside breast milk (25.47% of all mothers).

**Table 2:** Formula use with or without breast milk

Type of Feeding	Count	Percent
Formula Alone	58	51.79
Formula with Breast Milk	54	48.21
Total	112	100

48 mothers (22.64%) could not use their entire six months of maternity leave [consisting of 66.67% in governmental and 33.33% in private organizations], and 164 (77.36%) had used their maternity leave completely. Out of 212 mothers, 46 (21.7%) had nurseries at their workplace, while 166 (78.3%) did not have a proper place to take care of their infants. Also, 50 mothers (23.59%) had a suitable physical space for milking pumping in their workplace while 162 (76.47%) did not. 72 mothers (33.96%) milked their breasts at their workplace. Table 3 presents the manner of milking:

**Table 3:** Manner of milking at workplace

Manner of Milking	Count	Percent
Manual	32	44.44
Hand-Operated Milking Device	38	52.78
Electronic Milking Device	2	2.78
Total	72	100

166 mothers (78.3%) had been trained for milking while 46 (21.7%) had received no education in this regard. 116 mothers (54.72%) worked more than 6 hours per day. 104 mothers (49.01%) could use breastfeeding breaks and the rest (54 mothers, 50.99%) could not leave their workplace for breastfeeding. Table 4 presents the frequency of formula use for different occupational factors:

**Table 4:** Count and percent of formula use in employed mothers for different occupational factors

Occupational Factors		Count	Formula Use Count	Formula Use Percent	P Value
Maternity leave	> 6 months	164	39	47.56	0.05
	< 6 months	48	17	70.83	
Nursery at workplace	Available	46	9	39.13	0.14
	Unavailable	166	47	56.63	
Suitable physical space for milking	Available	50	7	28	0.004
	Unavailable	162	49	59.26	
Working hours	> 6 hours	116	35	60.34	0.088
	< 6 hours	96	21	43.75	
Training for breastfeeding and milking	Received	166	44	53.01	0.943
	Not received	46	12	52.17	
Possibility of breastfeeding breaks	Available	104	26	50	0.567
	Unavailable	108	30	55.56	

Significance: P < 0.05

## 4. Discussion

As Table 4 indicates, there is a significant relationship between formula use and maternity leave more than 6 months, lack of a suitable physical space for milk pumping.

Statistics indicate the rising number of infants who are breastfed for at least a year, as well as a reduction in imported baby formulas (Sadrizadeh, 2001); nevertheless, maternal occupation has been mentioned as an essential factor in cases of breastfeeding failure (Mohammadi et al., 2004; Mohsenzadeh & Mardani, 2008; Ghotbi, 2008; Khayyati, 2008; Abedzadeh et al., 2006; Ghaed Mohammadi et al., 2005; Akaberian & Dianat, 2004). The present study deals with the impact of occupational factors on breastfeeding of employed mothers. Our findings indicate that employed mothers with infants aged 6-12 months used formulas in 52.83% of cases, with breast milk completely discontinued in 27.36% of cases. In a study by Amin et al. (2011) in Malaysia, 51% of employed mothers, and in a study by Chuang et al. (2010) in Thailand 87.3% of mothers discontinued breastfeeding at one year, which are higher figures compared to our findings (27.36%). According to the Ministry of Health & Medical Education (2005), 9.5% of infants aged less than one year were formula fed and the mean age of discontinuing breastfeeding was 10.7 months. The discrepancy is caused by the fact that the Ministry of Health included all mothers (employed or home makers) and infants from birth to one year of age, while we have only recruited employed mothers with infants aged 6-12 months. However, the mean age of discontinuing breastfeeding which fall in the second 6 months of life (10.7 months) warrants closer investigation of influencing factors, including maternal occupation.

In our study, 22.64% of mothers returned to work sooner before the legal termination of their maternity leave. It is interesting that 66.67% of these women were employed in the governmental sector, while the executive mandate of the Law for Promotion of Breastfeeding clearly defines the maternity leave to be 6 months (According to the Ministry of Health & Medical Education, 2005). In this study, 70.83% of mothers who ended their maternity leave prematurely used formula, which is significantly higher than those who used 6 months or more of maternity leave (p=.005). It indicates relationship between early return to work and increased risk of formula use. Similarly, in the study conducted by Chuang et al. (2010) in Thailand, premature return to work (one month after labor) was associated with decreased duration of breastfeeding (from 73.2% to 65.1%) and the authors concluded that early return to work by mother is a challenge against duration and continuation of breastfeeding. Ogbuana et al. (2011) reported that mothers who returned to workplace after 13 weeks of leave were 2.54 times more likely to have an extended breastfeeding duration, corroborating our findings.

In our study, 78.3% of mothers did not have access to nursery at their workplace, and 56.63% of these women used formula which is not significantly higher than those who had nurseries in their workplace (p=0.14). Moreover, we found that there is no suitable physical space for breast milk pumping in 76.47% of cases, and 60.5% of these women used formula to feed their child, significantly higher than those women who had access to a suitable physical space for breast milk pumping in their workplace (p=0.004). A study in Brazil by Murais et al. (2011) reported unsupportive workplace as a challenge for breastfeeding employed women and highlighted the need for facilities to care for infants, milking and milk preservation. Amin et al. (2011) in Malaysia also mention the need for facilities such as milking room and refrigerators as a means for resolving the problems of breastfeeding mothers.

In the present study, 54.72% of mothers worked over 6 hours per day, and they used formula in 60.34% of cases, not significantly higher than those who worked less than six hours per day (p=0.088). Mandal et al. (2010) found an inverse relationship between working hours and duration of breastfeeding. Ogbuana et al. (2011) reported similar results in America.

In the present study, 78.3% of mothers had been trained for breastfeeding and milking, indicating the efforts of family health education units all over the country. Among untrained mothers (21.7%), 55.56% used formula which was not significantly higher compared to the trained group (p=0.943).

In this study, 50.99% of mothers could not leave their workplace to breastfeed their infants during working hours, and 55.56% of them used formula, not significantly higher than those who were allowed breastfeeding breaks (p=0.567).

Considering all that has been said above, maternity leave shorter than 6 months, lack of suitable physical space for breast milk pumping, all affect the success of breastfeeding. We recommend future studies to be conducted prospectively at intervals of 6, 9 and 12 months to assess the success of breastfeeding, as it is conceivable that mothers who only use their own milk and supplemental food for now, may resort to formulas with increasing problems posed by their working conditions.

In line with the findings of the present study, we recommend improved monitoring of governmental and private organizations in terms of full maternity leaves by Ministry of Health & Medical Education, preparation of nurseries with suitable physical space for breast milk pumping in workplaces, and part-time employment of mothers during breastfeeding.

## References

[ref1] Abedzadeh M, Saberi F, Sadat Z (2006). Quality of nutrition and factors related to it in 4.5-year old babies of Kashan. Feyz.

[ref2] Akaberian Sh, Dianat M (2004). Evaluation of factors influencing on non-exclusive breast feeding during the first six months of life in Bushehr port using focus group discussion. Tebe Jonoub.

[ref3] Amin R. M, Said Z. M, Sutan R, Shah S. A, Darus A, Shamsuddin K. H (2011). Work related determinants of breastfeeding discontinuation among employed mothers in Malaysia. International Breastfeeding Journal.

[ref4] Children office, Family & society health office, Health Undersecretary, Ministry of health and medical education (2002). Breastfeeding guide for employed mothers. Tehran: Ministry of health and medical education.

[ref5] Children office, Family & society health office, Health Undersecretary, Ministry of health and medical education (2007). Promotion breastfeeding & Protection of mothers during breastfeeding period law, Executive rule.

[ref6] Chuang C. H, Chang P. J, Chen Y. C, Hsieh W. S, Hurng B. S, Lin S. J (2010). Maternal Return to Work and Breastfeeding: A Population-Based Cohort Study. International Journal of Nursing Study.

[ref7] Family Health & Population Office, Ministry of Health & Medical Education (2005). Integrated Monitoring Evaluation System (IMES).

[ref8] Ghaed Mohammadi Z, Zafarmand M. H, Heidari G. H, Anaraki A, Dehghan A (2005). Determination of effective factors in breast-feeding continuity for infants less than 1 year old in urban area of Bushehr province. Tebe Jonoub.

[ref9] Ghotbi F (2008). Comparison between breastfeeding in employed and un employed women. Journal of the Faculty of Medicine.

[ref10] Hajian K. A (2002). Surveying the patterns of breast feeding in Babol. Journal of the Faculty of Medicine.

[ref11] Khayyati F (2008). An investigation into the reasons of terminating breastfeeding before the age of two. Scientific Journal of GUMS.

[ref12] Mamoori Gh, Hashemi Z (2002). Barrasiye vijegihaye shoghli va vazeeyate shirdehi ye madarane shaghel e shahre Zahedan. Iranian Journal of Nursing and Midwifery Research.

[ref13] Mandal B, Roe B. E, Fein S. B (2010). The Differential Effect of Full-time and Part-time Work Status on Breastfeeding. Health Policy.

[ref14] Mohammadi M. A, Dadkhah B, Mozaffari N (2004). Moroori bar tahghighate anjam shodeh dar zamineye elale adame movaffaghiyate shirdehi dar madarane shirdeh va avamele jesmi ravani va ejtemaeeye moasser bar an. Iranian Journal of Health & Care.

[ref15] Mohsenzadeh A, Mardani M (2008). Evaluation of exclusively breastfeeding failure reasons. YAFTE-E.

[ref16] Morais A. M. B, Machado M. M. T, Aquino P. S, Almeida M. I (2011). Breastfeeding Esperiences of Women Who Work at a Textile Industry from Ceara, Brazil. Revista Brasileira de Enfermagem.

[ref17] Ogbuana C, Glover S, Probst J, Liu J, Hussey J (2011). The Effect of Maternity Leave Length and Time of Return to Work on Breastfeeding. Pediatrics.

[ref18] Ogbuana C, Glover S, Probst J, Liu J, Hussey J (2011). Balancing Work and Family: Effect of Employment Characteristics on Breastfeeding. Human Lactation.

[ref19] Payne D, Nicholls D. A (2010). Managing Breastfeeding and work: a Foucauldian Secondary Analysis. Journal of Advanced Nursing.

[ref20] Rojjanasrirat W, Sousa V. D (2010). Perception of Breastfeeding and Planned Return to Work or School Among Low-income Pregnant Women in the USA. Journal of Clinical Nursing.

[ref21] Sadrizadeh B (2001). Iranian Health Situation and Trend in the Islamic Republic of Iran. J publ. Health.

[ref22] Schanler R. J (2006). Breastfeeding Handbook for Physicians.

[ref23] Unicef (2011). Iran, Islamic Republic of, Statistics: Nutrition. http://www.unicef.org/infobycountry/iran_statistics.html#0.

